# Supplementary material for: The Influence of Environmental Polycyclic Aromatic Hydrocarbons (PAHs) Exposure on DNA Damage among School Children in Urban Traffic Area, Malaysia
Source: Int J Environ Res Public Health. 2022 Feb 15;19(4):2193. doi: 10.3390/ijerph19042193 (PMC8872109; doi:10.3390/ijerph19042193)
Supplement: Supplementary file 1 [file ijerph-19-02193-s001.zip › Supplementary S1. Questionnaire.pdf]

Supplementary S1

ID Responden (diisi oleh penyelidik)

BORANG SOAL SELIDIK

BAHAGIAN A: BUTIRAN DIRI (KANAK-KANAK DAN WARIS)

A1. BUTIRAN DIRI KANAK-KANAK

1. Jantina : a. Lelaki ☐ b. Perempuan ☐
2. Tarikh Lahir : ___________________________ (Hari/Bulan/Tahun)
3. Sudah berapa lama tinggal di alamat sekarang : ☐☐ tahun ☐☐ bulan
4. Tahun berdaftar di sekolah ini : ☐☐☐☐
5. Bangsa: __________________

A2. BUTIRAN DIRI WARIS (sila isikan yang berkenaan untuk yang masih hidup sahaja)

1. Pendidikan Bapa/Penjaga Lelaki
   - a. Tidak Bersekolah ☐
   - b. Sekolah Rendah ☐
   - c. Tingkatan 3 ☐
   - d. Tingkatan 5 ☐
   - e. Sijil ☐
   - f. STPM/Diploma ☐
   - g. Ijazah ☐
   - h. Sarjana ☐
   - i. Doktor Falsafah ☐
   - j. Lain-lain. Nyatakan ____________ ☐
2. Pendidikan Ibu/Penjaga Perempuan
   - a. Tidak Bersekolah ☐
   - b. Sekolah Rendah ☐
   - c. Tingkatan 3 ☐
   - d. Tingkatan 5 ☐
   - e. Sijil ☐
   - f. STPM/Diploma ☐
   - g. Ijazah ☐
   - h. Sarjana ☐
   - i. Doktor Falsafah ☐
   - j. Lain-lain. Nyatakan _____________ ☐
3. Pekerjaan Bapa/Penjaga Lelaki
   - a. Majikan ☐
   - b. Kakitangan Kerajaan ☐
   - c. Kakitangan Swasta ☐
   - d. Bekerja Sendiri ☐
   - e. Tidak Bekerja ☐
   - f. Pesara ☐
4. Pekerjaan Ibu/Penjaga Perempuan
   - a. Majikan ☐
   - b. Kakitangan Kerajaan ☐
   - c. Kakitangan Swasta ☐
   - d. Bekerja Sendiri ☐
   - e. Tidak Bekerja ☐
   - f. Pesara ☐
5. Anggaran pendapatan kasar sebulan
   - a. Bapa/Penjaga Lelaki RM____________
   - b. Ibu/Penjaga Perempuan RM____________

BAHAGIAN B: STATUS KESIHATAN

	YA	TIDAK
1. Adakah kanak-kanak ini menghidap penyakit kronik?<br>**Jika YA**, sila nyatakan: _________________________________		
Adakah kanak-kanak ini pernah menjalani rawatan di bawah dalam tempoh berkenaan:		
a) Kemoterapi (dalam tempoh 6 bulan lepas)		
b) Radioterapi (dalam tempoh 6 bulan lepas)		

c) X-ray (dalam tempoh 3 bulan lepas) ☐ ☐

d) Lain-lain. Nyatakan: _________________________________ ☐ ☐

3. Adakah ahli keluarga terdekat (ibu, bapa, adik-beradik, atuk, nenek) menghidap penyakit kronik? **Jika YA**, sila nyatakan: ☐ ☐

Penyakit: ____ Siapa: ____

BAHAGIAN C: SEJARAH KESIHATAN RESPIRATORI

C1. BATUK

		YA	TIDAK
1.	Adakah anak anda selalu mengalami batuk berserta selesema?		

C2. KAHAK

		YA	TIDAK
1.	Adakah anak anda kerap mengalami sesak nafas atau mengeluarkan kahak berserta selesema?		

C3. DADA BERBUNYI

		YA	TIDAK
1.	Adakah anak anda selalu mengalami masalah pernafasan berbunyi di bahagian dada?		

C4. KESAKITAN DADA

		YA	TIDAK
1.	Sejak 3 tahun lepas, adakah anak anda pernah mengalami kesesakan bahagian dada yang menghalang anak anda daripada melakukan aktiviti biasa selama 3 hari?		

C5. ALERGI/ALAHAN

		YA	TIDAK
1.	Adakah doktor pernah mengatakan anak anda mengalami alahan kepada debu?		

BAHAGIAN D: PENDEDAHAN KEPADA ASAP ROKOK

		YA	TIDAK
1.	Adakah ahli keluarga yang tinggal di rumah anda/pengasuh anak anda seorang perokok? Jika YA, sila nyatakan siapa: ____________________		

BAHAGIAN E: PERSEKITARAN RUMAH

1. Di manakah lokasi perumahan anda?
   a) Bandar ☐ b) Sub-bandar ☐ c) Luar Bandar ☐

2. Kanak-kanak ini tidur/tinggal di dalam bilik
   a) sendiri ☐ c) berkongsi dengan 1 orang ☐
   b) berkongsi dengan 2 orang ☐ d) berkongsi dengan ≥ 3 orang ☐

3. Apakah bahan api yang digunakan untuk memasak di dalam rumah anda?
   a) Elektrik ☐ c) Minyak Tanah ☐ e) Arang ☐
   b) Gas ☐ d) Kayu Api ☐ f) Lain-lain. Nyatakan: ________________ ☐

4. Berapa kali dalam sehari anda menggunakan bahan api di atas untuk memasak? _____ kali

5. Semasa memasak, adakah anda membuka tingkap atau pintu untuk membenarkan Ya ☐ Tidak ☐

pengaliran udara di dalam rumah?

6. Apakah alat yang digunakan untuk menyejukkan udara di dalam rumah?
a) Penyaman Udara ☐ b) Kipas ☐ c) Lain-lain. Nyatakan: ___________

7. Adakah anda menggunakan bahan tertentu untuk mengelakkan serangan nyamuk? Ya ☐ Tidak ☐

8. **Jika YA**, apakah jenis yang selalu digunakan?
a) Lingkaran biasa ☐
b) Elektrik ☐
c) Semburan aerosol ☐
d) Lain-lain. Nyatakan ________________ ☐

9. Lokasi rumah dari jalan utama:
a) < 100 m dari jalan utama ☐
b) 100 – 199 m dari jalan utama ☐
c) 200 – 299 m dari jalan utama ☐
d) 300 – 399 m dari jalan utama ☐
e) 400 – 499 m dari jalan utama ☐
f) ≥ 500 m dari jalan utama ☐

10. Lokasi rumah dari lebuhraya:
a) < 1 km dari lebuhraya ☐
b) 1 – 1.9 km dari lebuhraya ☐
c) 2 – 2.9 km dari lebuhraya ☐
d) 3 – 3.9 km dari lebuhraya ☐
e) 4 – 4.9 km dari lebuhraya ☐
f) ≥ 5 km dari lebuhraya ☐

11. Lokasi rumah dari kilang:
a) < 2.5 km dari kilang ☐
b) 2.5 – 5 km dari kilang ☐
c) ≥ 5 km dari kilang ☐

12. Adakah anda kerap melakukan pembakaran terbuka di luar rumah seperti membakar daun-daun kering? Ya ☐ Tidak ☐

13. Apakah kenderaan yang paling kerap digunakan oleh anak anda untuk ke sekolah?
a) Kereta ☐
b) Bas ☐
c) Basikal ☐
d) Motosikal ☐
e) Van ☐
f) Berjalan kaki ☐

14. Berapa kerapkah anak anda makan daging yang dipanggang? ___________

15. Berapa kerapkah anak anda makan buah-buahan dan sayur sayuran ? __________

16. Adakah anak anda mengambil makanan tambahan seperti vitamin atau jus? ____________
