# Supplementary material for: The Influence of Environmental Polycyclic Aromatic Hydrocarbons (PAHs) Exposure on DNA Damage among School Children in Urban Traffic Area, Malaysia
Source: Int J Environ Res Public Health. 2022 Feb 15;19(4):2193. doi: 10.3390/ijerph19042193 (PMC8872109; doi:10.3390/ijerph19042193)
Supplement: Supplementary file 1 [file ijerph-19-02193-s001.zip › Supplementary S2. Distributions of PAHs species (ng m-3) in indoor and outdoor PM2.5 samples.pdf]

Supplementary S2. Distributions of PAHs species (ng m^{-3}) in indoor and outdoor $PM_{2.5}$ samples.

Compound	H1		H2		H3		H4		L1		L2		L3		L4	
	Indoor	Outdoor	Indoor	Outdoor	Indoor	Outdoor	Indoor	Outdoor	Indoor	Outdoor	Indoor	Outdoor	Indoor	Outdoor	Indoor	Outdoor
**Total PAHs**	**5.58 ± 4.72**	**5.76 ± 2.20**	**4.19 ± 0.91**	**4.40 ± 1.79**	**4.86 ± 3.17**	**5.69 ± 3.22**	**4.65 ± 1.39**	**4.96 ± 1.96**	**3.48 ± 3.14**	**3.79 ± 1.93**	**3.69 ± 3.19**	**3.72 ± 2.53**	**1.25 ± 0.83**	**1.36 ± 0.69**	**2.26 ± 1.32**	**2.63 ± 1.96**
ACY	0.08 ± 0.11	0.46 ± 0.15	0.26 ± 0.10	0.38 ± 0.11	0.35 ± 0.26	0.64 ± 0.12	0.32 ± 0.18	0.31 ± 0.06	0.45 ± 0.41	0.45 ± 0.29	0.44 ± 0.59	0.49 ± 0.48	0.23 ± 0.20	0.17 ± 0.09	0.27 ± 0.18	0.22 ± 0.15
ACP	0.26 ± 0.37	0.88 ± 0.39	0.62 ± 0.24	0.48 ± 0.42	0.60 ± 0.45	0.75 ± 0.59	0.56 ± 0.33	0.41 ± 0.16	0.91 ± 0.87	0.75 ± 0.45	0.84 ± 0.75	0.81 ± 0.69	LOD	0.19 ± 0.14	0.35 ± 0.23	0.54 ± 0.51
FLR	0.19 ± 0.23	0.43 ± 0.16	0.34 ± 0.07	0.42 ± 0.02	0.31 ± 0.24	0.16 ± 0.23	0.30 ± 0.11	0.56 ± 0.16	0.55 ± 0.49	0.80 ± 0.20	0.53 ± 0.75	0.83 ± 0.22	LOD	LOD	0.45 ± 0.26	0.32 ± 0.32
PHE	0.01 ± 0.00	0.05 ± 0.02	0.03 ± 0.00	0.03 ± 0.02	0.04 ± 0.03	0.07 ± 0.04	0.03 ± 0.01	0.03 ± 0.00	0.04 ± 0.03	0.04 ± 0.02	0.05 ± 0.02	0.05 ± 0.01	0.04 ± 0.02	0.04 ± 0.01	0.02 ± 0.01	0.02 ± 0.01
ANT	0.11 ± 0.15	0.15 ± 0.12	0.13 ± 0.14	0.15 ± 0.18	0.14 ± 0.18	0.03 ± 0.04	0.15 ± 0.18	0.10 ± 0.11	0.06 ± 0.01	0.08 ± 0.09	0.10 ± 0.14	0.10 ± 0.12	0.04 ± 0.02	0.03 ± 0.02	0.13 ± 0.16	0.18 ± 0.11
FLT	0.06 ± 0.04	0.15 ± 0.04	0.14 ± 0.03	0.13 ± 0.07	0.12 ± 0.12	0.27 ± 0.24	0.15 ± 0.07	0.14 ± 0.05	0.13 ± 0.11	0.13 ± 0.11	0.15 ± 0.10	0.13 ± 0.12	0.06 ± 0.03	0.06 ± 0.01	0.04 ± 0.04	0.10 ± 0.06
PYR	0.24 ± 0.20	0.42 ± 0.04	0.21 ± 0.02	0.31 ± 0.10	0.25 ± 0.16	0.38 ± 0.11	0.17 ± 0.03	0.39 ± 0.22	0.09 ± 0.09	0.16 ± 0.07	0.21 ± 0.16	0.19 ± 0.13	0.10 ± 0.06	0.13 ± 0.11	0.16 ± 0.08	0.11 ± 0.06
BaA	0.11 ± 0.09	0.09 ± 0.06	0.09 ± 0.01	0.08 ± 0.00	0.08 ± 0.08	0.20 ± 0.16	0.10 ± 0.03	0.12 ± 0.05	0.06 ± 0.05	0.06 ± 0.05	0.06 ± 0.05	0.04 ± 0.06	0.02 ± 0.01	0.02 ± 0.01	0.02 ± 0.02	0.06 ± 0.04
CYR	0.13 ± 0.08	0.12 ± 0.08	0.15 ± 0.03	0.11 ± 0.02	0.10 ± 0.10	0.27 ± 0.24	0.16 ± 0.10	0.15 ± 0.06	0.08 ± 0.07	0.08 ± 0.07	0.09 ± 0.06	0.07 ± 0.10	0.03 ± 0.02	0.02 ± 0.01	0.02 ± 0.02	0.09 ± 0.06
BaP	0.57 ± 0.29	0.29 ± 0.15	0.41 ± 0.03	0.20 ± 0.08	0.28 ± 0.17	0.26 ± 0.14	0.47 ± 0.03	0.23 ± 0.10	0.05 ± 0.09	0.11 ± 0.07	0.10 ± 0.08	0.02 ± 0.03	0.21 ± 0.12	0.23 ± 0.07	0.15 ± 0.17	0.13 ± 0.11
BbF	0.68 ± 0.48	0.50 ± 0.17	0.26 ± 0.05	0.35 ± 0.21	0.44 ± 0.28	0.43 ± 0.18	0.26 ± 0.01	0.34 ± 0.11	0.14 ± 0.13	0.21 ± 0.03	0.19 ± 0.01	0.19 ± 0.00	0.09 ± 0.09	0.10 ± 0.05	0.15 ± 0.01	0.15 ± 0.08
BkF	1.84 ± 1.64	1.04 ± 0.40	0.58 ± 0.12	0.71 ± 0.23	0.92 ± 0.49	0.94 ± 0.54	0.64 ± 0.08	0.87 ± 0.35	0.38 ± 0.33	0.39 ± 0.21	0.34 ± 0.28	0.34 ± 0.26	0.19 ± 0.06	0.13 ± 0.05	0.19 ± 0.00	0.34 ± 0.28
BgP	0.22 ± 0.19	0.17 ± 0.09	0.17 ± 0.01	0.15 ± 0.03	0.18 ± 0.13	0.21 ± 0.10	0.29 ± 0.12	0.21 ± 0.09	0.06 ± 0.05	0.07 ± 0.02	0.07 ± 0.01	0.03 ± 0.05	0.03 ± 0.02	0.04 ± 0.02	0.04 ± 0.03	0.06 ± 0.02
DhA	0.40 ± 0.38	0.39 ± 0.04	0.21 ± 0.05	0.32 ± 0.21	0.34 ± 0.16	0.46 ± 0.25	0.29 ± 0.00	0.33 ± 0.12	0.24 ± 0.21	0.21 ± 0.13	0.27 ± 0.09	0.24 ± 0.09	0.11 ± 0.11	0.06 ± 0.06	0.11 ± 0.05	0.13 ± 0.11
IcP	0.70 ± 0.47	0.63 ± 0.29	0.61 ± 0.01	0.59 ± 0.09	0.71 ± 0.32	0.63 ± 0.25	0.78 ± 0.11	0.75 ± 0.33	0.23 ± 0.20	0.25 ± 0.11	0.25 ± 0.11	0.19 ± 0.17	0.09 ± 0.07	0.14 ±0.03	0.16 ± 0.05	0.17 ± 0.06

LOD=Below limit of detection
