# Supplementary material for: The Influence of Environmental Polycyclic Aromatic Hydrocarbons (PAHs) Exposure on DNA Damage among School Children in Urban Traffic Area, Malaysia
Source: Int J Environ Res Public Health. 2022 Feb 15;19(4):2193. doi: 10.3390/ijerph19042193 (PMC8872109; doi:10.3390/ijerph19042193)
Supplement: Supplementary file 1 [file ijerph-19-02193-s001.zip › Supplementary S4. & Supplementary S5. TEQ of PAHs compounds in outdoor and indoor air of studied primary schools.pdf]

Supplementary S4. TEQ of PAHs compounds in outdoor air of studied primary schools

Compounds	TEFs	H1	H2	H3	H4	L1	L2	L3	L4
ACY	0.001	0.00	0.00	0.00	0.00	0.00	0.00	0.00	0.00
ACP	0.001	0.00	0.00	0.00	0.00	0.00	0.00	0.00	0.00
FLU	0.001	0.00	0.00	0.00	0.00	0.00	0.00	0.00	0.00
PHE	0.001	0.00	0.00	0.00	0.00	0.00	0.00	0.00	0.00
ANT	0.01	0.00	0.00	0.00	0.00	0.00	0.00	0.00	0.00
FLA	0.001	0.00	0.00	0.00	0.00	0.00	0.00	0.00	0.00
PYR	0.001	0.00	0.00	0.00	0.00	0.00	0.00	0.00	0.00
BaA	0.1	0.01	0.01	0.02	0.01	0.01	0.00	0.00	0.01
CYR	0.01	0.00	0.00	0.00	0.00	0.00	0.00	0.00	0.00
BaP	1	0.29	0.20	0.26	0.23	0.11	0.02	0.23	0.13
BbF	0.1	0.05	0.04	0.04	0.03	0.21	0.19	0.10	0.15
BkF	0.1	0.10	0.07	0.09	0.09	0.04	0.03	0.01	0.03
BgP	0.01	0.00	0.00	0.00	0.00	0.00	0.00	0.00	0.00
DhA	5	1.96	1.59	2.29	1.67	1.07	1.21	0.31	0.67
IcP	0.1	0.06	0.06	0.06	0.08	0.02	0.02	0.01	0.02
∑TEQ (ng m^{-3})		**2.48**	**1.96**	**2.77**	**2.12**	**1.46**	**1.48**	**0.67**	**1.01**

Supplementary S5. TEQ of PAHs compounds in indoor air of studied primary schools

Compounds	TEFs	H1	H2	H3	H4	L1	L2	L3	L4
ACY	0.001	0.00	0.00	0.00	0.00	0.00	0.00	0.00	0.00
ACP	0.001	0.00	0.00	0.00	0.00	0.00	0.00	0.00	0.00
FLU	0.001	0.00	0.00	0.00	0.00	0.00	0.00	0.00	0.00
PHE	0.001	0.00	0.00	0.00	0.00	0.00	0.00	0.00	0.00
ANT	0.01	0.00	0.00	0.00	0.00	0.00	0.00	0.00	0.00
FLA	0.001	0.00	0.00	0.00	0.00	0.00	0.00	0.00	0.00
PYR	0.001	0.00	0.00	0.00	0.00	0.00	0.00	0.00	0.00
BaA	0.1	0.01	0.01	0.01	0.01	0.01	0.01	0.00	0.00
CYR	0.01	0.00	0.00	0.00	0.00	0.00	0.00	0.00	0.00
BaP	1	0.57	0.41	0.28	0.47	0.05	0.10	0.21	0.15
BbF	0.1	0.03	0.01	0.02	0.01	0.14	0.19	0.09	0.15
BkF	0.1	0.18	0.06	0.09	0.06	0.04	0.03	0.02	0.02
BgP	0.01	0.00	0.00	0.00	0.00	0.00	0.00	0.00	0.00
DhA	5	2.00	1.03	1.71	1.44	1.20	1.33	0.53	0.53
IcP	0.1	0.07	0.06	0.07	0.08	0.02	0.03	0.01	0.02
∑TEQ (ng m^{-3})		**2.87**	**1.59**	**2.19**	**2.08**	**1.47**	**1.69**	**0.86**	**0.87**
