# Supplementary material for: The Influence of Environmental Polycyclic Aromatic Hydrocarbons (PAHs) Exposure on DNA Damage among School Children in Urban Traffic Area, Malaysia
Source: Int J Environ Res Public Health. 2022 Feb 15;19(4):2193. doi: 10.3390/ijerph19042193 (PMC8872109; doi:10.3390/ijerph19042193)
Supplement: Supplementary file 1 [file ijerph-19-02193-s001.zip › Supplementary S6. Predictor factors of DNA damage (Tail moment) among children.pdf]

Supplementary S6. Predictor factors of DNA damage (Tail moment) among children

**Variables**	***B* (95% CI)**	**β**	***p*-value**
Indoor total PAHs [a]	0.063 (0.026, 0.100)	0.220	0.001*
Indoor carcinogen PAHs [a]	0.080 (0.033, 0.127)	0.217	0.001*
Indoor non carcinogen PAHs [a]	0.047 (-0.019, 0.113)	0.092	0.165
Outdoor total PAHs [a]	0.051 (0.005, 0.097)	0.143	0.031*
Outdoor carcinogen PAHs [a]	0.098 (0.025, 0.171)	0.173	0.009*
Outdoor non carcinogen PAHs [a]	0.040 (-0.045, 0.125)	0.062	0.354
Age [a]	0.088 (-0.019, 0.195)	0.107	0.108
Gender [b]	-0.025 (-0.197, 0.147)	-0.019	0.773
BMI [a]	-0.007 (-0.025, 0.011)	-0.054	0.419
ETS [b]	0.061 (-0.116, 0.238)	0.045	0.495
Distance from main road [b]	0.088 (-0.149, 0.326)	0.049	0.465
Distance from highway [b]	0.202 (0.022, 0.382)	0.146	0.028*
Transportation to school [b]	0.021 (-0.297, 0.338)	0.008	0.899
Grilled food [b]	0.068 (-0.159, 0.296)	0.039	0.554
Supplement intake [b]	-0.026 (-0.198, 0.145)	-0.020	0.763
Fruit consumption [b]	0.188 (-0.052, 0.427)	0.102	0.123

N=228
Simple linear regression (Method: Enter)
[a] Continuous variable, [b] Categorical variable
B = unstandardised coefficient, β=standardised coefficient
*Significant at $p<0.05$
