# Supplementary figures and images for: The Influence of Environmental Polycyclic Aromatic Hydrocarbons (PAHs) Exposure on DNA Damage among School Children in Urban Traffic Area, Malaysia
Source: Int J Environ Res Public Health. 2022 Feb 15;19(4):2193. doi: 10.3390/ijerph19042193 (PMC8872109; doi:10.3390/ijerph19042193)

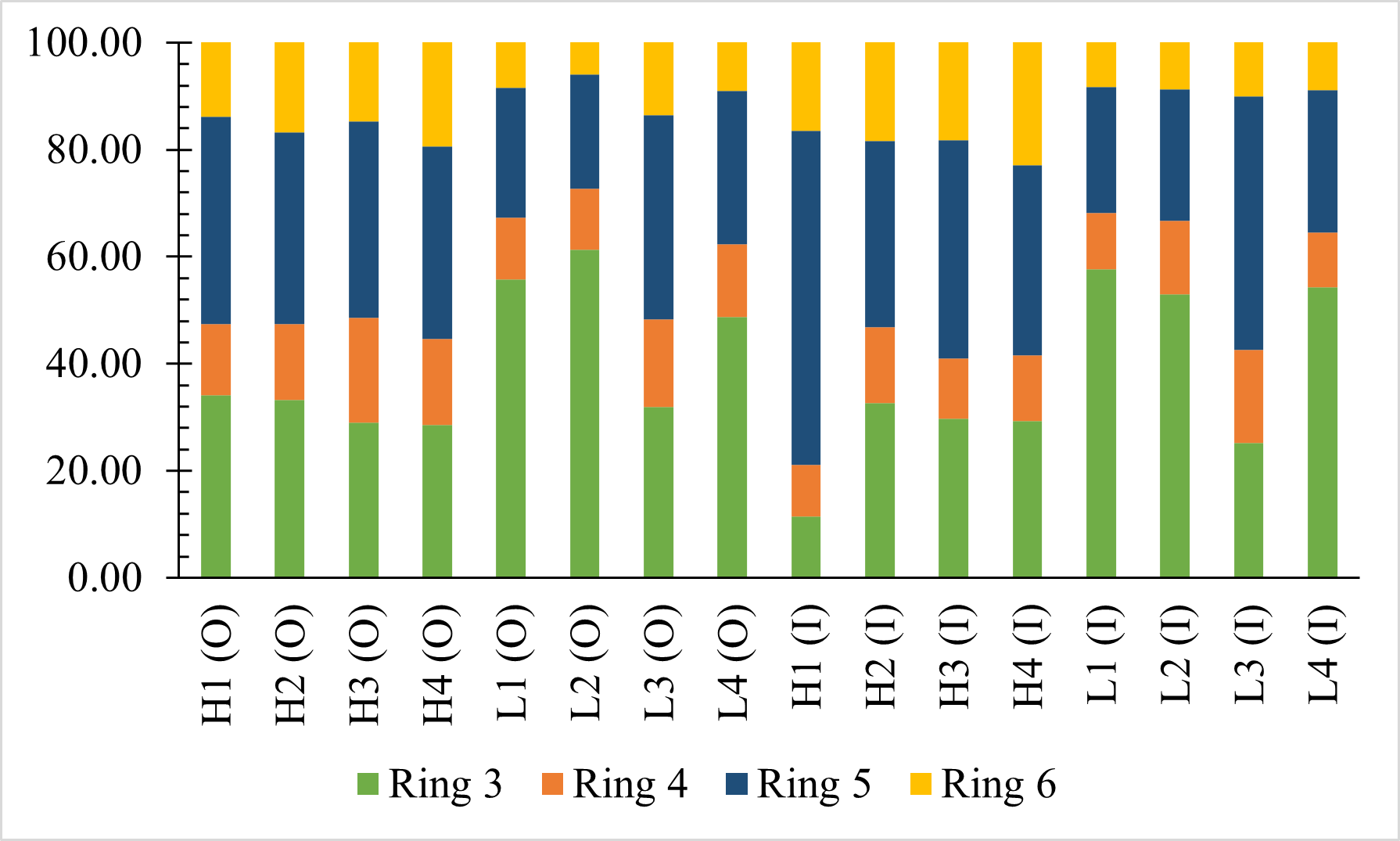

Supplement: Supplementary file 1 [file ijerph-19-02193-s001.zip › Supplementary S3. Percentage distribution of outdoor and indoor PAHs based on number of rings.png]
